# A CT-based radiomics model for noninvasive prediction of MMP9 expression and prognostic evaluation in renal cell carcinoma

**DOI:** 10.1097/MD.0000000000048992

**Published:** 2026-06-05

**Authors:** Sijia Sun, Jin Zhang

**Affiliations:** a Department of Postgraduate Work, Xi’an Medical University, Xi’an, Shaanxi Province, China; b Department of Traditional Chinese Medicine, Tangdu Hospital, Fourth Military Medical University, Xi’an, Shaanxi Province, China; c Key Laboratory of Integrated Traditional Chinese and Western Medicine Tumor Diagnosis and Treatment in Shaanxi Province, Xi’an, Shaanxi Province, China.

**Keywords:** CT, MMP9, radiomics, renal cell carcinoma, tumor microenvironment

## Abstract

Renal cell carcinoma (RCC) represents one of the most common malignant tumors of the urinary system, characterized by high heterogeneity and aggressiveness. Studies indicate that matrix metalloproteinase-9 (MMP9) plays a critical role in the invasion, metastasis, and prognosis of RCC, and is recognized as a potential molecular biomarker and therapeutic target. This study aims to develop a radiomics-based prediction model for noninvasive identification of MMP9 expression levels in RCC patients and to assess its clinical relevance in patient prognosis. This retrospective study utilized enhanced computed tomography images and clinical data from The Cancer Genome Atlas database. We manually delineated volumes of interest, extracted radiomic features, and performed standardization procedures. Key features were selected using minimum redundancy maximum relevance and recursive feature elimination algorithms, followed by the construction of a radiomics model for predicting MMP9 expression through the gradient boosting machine algorithm. Survival analysis was performed using Kaplan–Meier curves and Cox proportional hazards regression models. Furthermore, immune cell infiltration analysis and enrichment analysis were employed to explore the underlying biological mechanisms associated with MMP9. MMP9 demonstrated independent prognostic value in RCC patients (hazard ratio = 1.447). The radiomics model incorporating 3 features, constructed using multiple machine learning algorithms, exhibited favorable predictive performance for MMP9 expression levels (area under the curve = 0.865). The radiomics score mapping MMP9 expression levels served as an independent risk predictor for RCC patients (hazard ratio = 2.287, 95% confidence interval = 1.025–5.103, *P* = .043). Furthermore, MMP9 correlates with pathways such as immune response regulation signals, carbon metabolism, and PI3K-Akt, as well as immune cell infiltration, including M2-type macrophages and regulatory T cells. MMP9 serves as an independent prognostic factor in RCC. The enhanced computed tomography radiomics model composed of 3 features can noninvasively predict MMP9 expression levels in RCC patients.

## 1. Introduction

Renal cell carcinoma (RCC) represents the primary type of kidney cancer, accounting for over 90% of all renal malignancies.^[1]^ RCC ranks as the 14th most commonly diagnosed cancer worldwide, with 434,840 new cases reported globally in 2022.^[2,3]^ According to the Global Burden of Disease data, the age-standardized incidence rate of kidney cancer exhibited a rising trend between 2000 and 2021, with an average annual percentage change of 0.15%.^[4]^ Despite advancements in imaging and diagnostics, RCC is often asymptomatic in its early stages, and approximately 30% of patients are diagnosed at an advanced stage.^[5]^ The 5-year survival rate for patients with early-stage localized kidney cancer exceeds 90%, while plummeting to <20% for advanced-stage patients, highlighting the critical importance of early screening and precision diagnosis and treatment.^[6]^

The current clinical management system for RCC relies on imaging evaluation combined with pathological diagnosis as the gold standard, with treatment strategies encompassing multimodal interventions including surgical resection, molecular targeted therapy, and immunotherapy.^[7,8]^ However, current clinical practice still lacks effective prognostic prediction models, making it difficult to accurately assess patients’ prognostic risk and treatment response. Matrix metalloproteinase-9 (MMP9), a key regulator of tumor invasion and metastasis, has been shown to promote RCC pathological progression by degrading the extracellular matrix and is significantly associated with patient overall survival (OS).^[9–11]^ Despite its biological relevance, few studies have systematically examined the relationship between MMP9 expression and RCC prognosis. Furthermore, existing detection techniques (e.g., peripheral blood cytokine assays, mRNA analysis of tissue samples, and immunohistochemistry of paraffin-embedded specimens) commonly exhibit limitations such as operator dependency, antibody specificity variations, and high costs. These limitations hinder their application in routine clinical settings and highlight the need for alternative noninvasive and reproducible approaches to assess MMP9 expression.

Notably, radiomics technology has demonstrated advantages in tumor heterogeneity assessment, microenvironment characterization, and treatment response prediction, thereby offering novel pathways for noninvasive biomarker development.^[12,13]^ This study integrated transcriptomic data from The Cancer Genome Atlas (TCGA) to investigate the relationship between MMP9 expression and RCC patient prognosis. Furthermore, based on computed tomography (CT) images retrieved from The Cancer Imaging Archive (TCIA), we employed the gradient boosting machine (GBM) algorithm to develop a prediction model for MMP9 expression, evaluating both its predictive performance and clinical utility. By establishing a noninvasive imaging biomarker, this study aims to advance individualized prognosis prediction and inform personalized treatment strategies in RCC.

## 2. Materials and methods

### 2.1. Data collection

**Figure 1.**
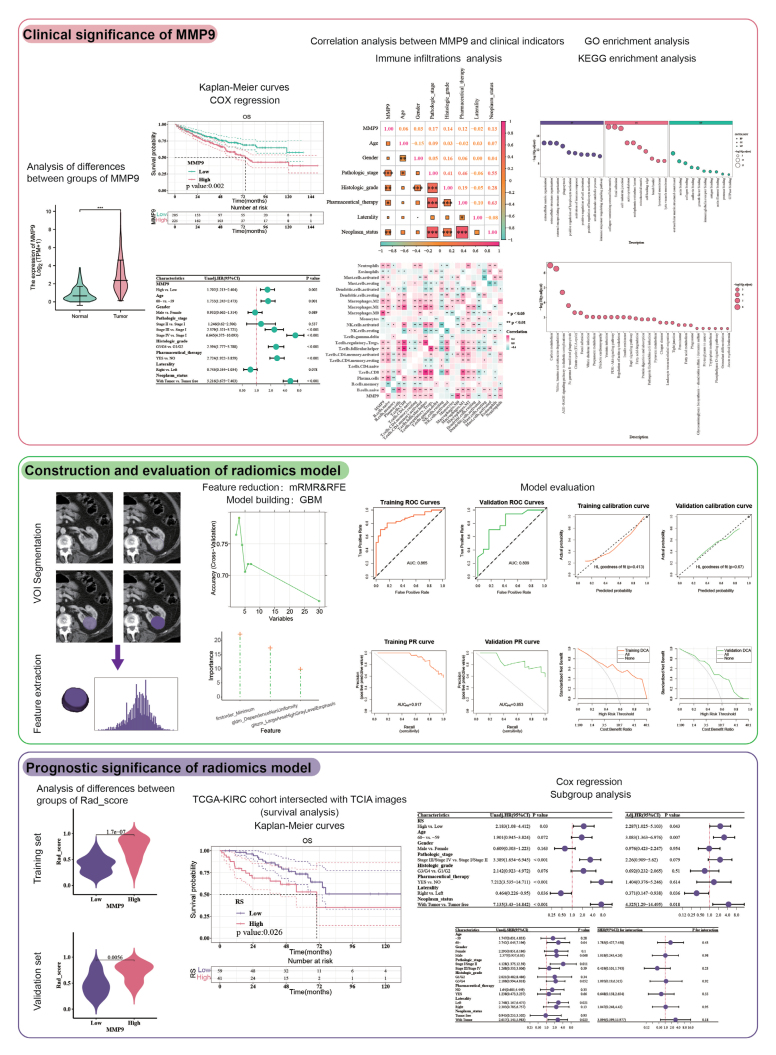
Flow chart of the study process. GBM = gradient boosting machine, GO = Gene Ontology, KEGG = Kyoto Encyclopedia of Genes and Genomes, KIRC = kidney renal clear cell carcinoma, MMP9 = matrix metalloproteinase-9, mRMR = minimum redundancy maximum relevance, RFE = recursive feature elimination, TCGA = The Cancer Genome Atlas, TCIA = The Cancer Imaging Archive, VOI = volumes of interest.

This study was approved by the Ethics Committee of Tangdu Hospital, Fourth Military Medical University. Figure 1 illustrates the research workflow. This study integrated contrast-enhanced CT imaging data from TCIA (https://www.cancerimagingarchive.net/) and corresponding transcriptomic and clinical data from TCGA (https://portal.gdc.cancer.gov/) to evaluate the prognostic value of MMP9 in clear cell RCC, construct a radiomics model for noninvasive prediction of MMP9 expression levels, and explore the prognostic significance of this prediction model for RCC patients. The inclusion and exclusion criteria were established as follows: for MMP9 prognostic analysis: screening first-diagnosed and treatment-naive clear cell adenocarcinoma patients; excluding samples with OS <30 days, missing clinical variables, non-primary solid tumors, and lacking RNA sequencing (RNA-seq) data; for radiomics model construction: eliminating samples that had undergone surgical treatment with poor imaging quality, and selecting samples with overlapping clinical data and RNA-seq data.

The included covariates comprised age (<60 years vs ≥60 years), gender (male vs female), tumor histological grade (G1/G2 vs G3/G4), tumor laterality (left vs right), pathological staging (stage I–IV), tumor status (tumor-free vs tumor-bearing), and pharmacotherapy status (yes vs no). The primary outcome measure was OS.

Since the TCGA and TCIA data used in this study contained de-identified patient information and were publicly available resources, the research did not require ethics review board approval, nor was patient informed consent required. Pharmacotherapy status was obtained from TCGA clinical annotations. However, because of the retrospective design and incomplete treatment timeline information, it was difficult to determine whether pharmacotherapy was strictly a pretreatment or posttreatment variable for every patient. Therefore, pharmacotherapy may reflect not only treatment exposure but also disease severity and clinical decision-making.

### 2.2. Survival analysis of MMP9

All eligible cases were divided into MMP9 high-expression and low-expression groups based on the optimal cutoff value determined by the R package “survminer.” Survival curves were plotted using the Kaplan–Meier method, and the log-rank test was employed to assess the statistical significance of survival differences between groups. The R package “survival” was used to perform survival analysis. Univariate and multivariate Cox proportional hazards regression models were applied to estimate hazard ratios (HRs) and 95% confidence intervals (CIs) for each variable. Subgroup analyses and interaction tests were further performed to evaluate the consistency of prognostic effects across different clinical strata. In addition, the Spearman rank correlation coefficient was employed to analyze the correlation between the primary variable, MMP9, and clinical tumor characteristics.

### 2.3. Exploring MMP9-associated biological processes based on transcriptome

RNA-seq data and corresponding clinical information of RCC patients were obtained from the TCGA database. Given the substantially fewer normal tissue samples than tumor samples in TCGA, this study additionally retrieved data from the Genotype-Tissue Expression database via the University of California, Santa Cruz XENA platform (https://xenabrowser.net/datapages/), obtained RNA-seq data in transcripts per million format preprocessed via the Toil pipeline,^[14]^ from which normal tissue data corresponding to RCC were extracted. RNA-seq data in transcripts per million reads format underwent log2 transformation followed by differential expression analysis across samples.

To explore the functions of potential target genes, functional enrichment analysis was performed: Gene Ontology (GO, including biological processes [BPs], cellular components, and molecular functions) and Kyoto Encyclopedia of Genes and Genomes (KEGG) enrichment analyses were conducted using the R package “clusterProfiler,” with the filtering threshold set at adjusted *P* value of <.05.^[15]^ The gene expression matrix of RCC samples was uploaded to the CIBERSORTx platform (https://cibersortx.stanford.edu/).^[16]^ Spearman correlation analysis was used to evaluate the association between MMP9 expression levels and the degree of immune cell infiltration.

### 2.4. Radiomics model construction

This study integrated TCGA bioinformatics data with TCIA imaging data, identifying 100 overlapping samples for analysis. Data were randomly partitioned into training and validation sets at a 7:3 ratio, with intergroup difference analysis performed on both cohorts. Qualified CT images obtained from TCIA were imported into 3D Slicer software (version 4.10.2; 3D Slicer Developer Community). All qualified CT images were imported into 3D Slicer. Radiologist A, with more than 5 years of experience in abdominal imaging, manually delineated the whole-tumor volumes of interest for all included patients before random allocation into the training and validation cohorts. To evaluate inter-observer reproducibility, Radiologist B independently re-delineated volumes of interest in 20 randomly selected cases, including patients from both the training and validation cohorts. The intraclass correlation coefficient was then calculated, and only radiomic features with an intraclass correlation coefficient (ICC) ≥ 0.75 were retained for further analysis.

Radiomic features were extracted using the PyRadiomics library (https://pyradiomics.readthedocs.io/), and features with high reproducibility (ICC ≥ 0.75) were retained for further analysis.^[17]^ Key features were further screened using the minimum redundancy maximum relevance algorithm and the recursive feature elimination method. A radiomics model was constructed based on the GBM algorithm, with the output probability value designated as the radiomics score (RS).

To evaluate model performance, receiver operating characteristic curves and precision-recall curves were plotted. Metrics including, the area under the curve (AUC), accuracy, sensitivity, specificity, positive predictive value, and negative predictive value, were calculated. The calibration capability of the model was evaluated through calibration curves and the Hosmer-Lemeshow goodness-of-fit test; predictive performance was comprehensively assessed using the Brier score; and decision curve analysis was plotted to quantify clinical net benefit. The DeLong test was employed to compare AUC values between the training set and validation set, thereby assessing model fitting.

### 2.5. Assessment of the prognostic value of the radiomics model in RCC patients

Based on the GBM radiomics model, RS were calculated for samples in the TCIA–TCGA intersection cohort. The Wilcoxon rank-sum test was used to analyze differences in RS between the gene high-expression and low-expression groups. After integrating RS with clinical data, the optimal cutoff value was determined using the “survminer” package, thereby stratifying patients into high-RS and low-RS groups. The prognostic value of RS was assessed by Kaplan–Meier analysis and Cox proportional hazards models. Subgroup analyses across clinical covariates were performed to explore the stability of RS-based risk stratification, and likelihood ratio tests were used to evaluate potential interaction effects between RS and clinical variables.

### 2.6. Statistical analysis

Continuous variables were summarized as mean ± standard deviation, while categorical variables were expressed as frequency and percentage (%). Group comparisons for categorical variables were performed using the chi-square test. Spearman correlation analysis was used to assess the correlation between the RS and other covariates as well as gene expression levels. Survival curves were generated using the Kaplan–Meier method, with between-group differences compared by the log-rank test. Survival analysis was conducted using the Cox proportional hazards regression model. All statistical tests were two-sided, with a *P* < .05 considered statistically significant. Statistical analyses were performed using R software (version 4.1.0; R Foundation for Statistical Computing). Visualization was conducted using the R packages “survival,” “forestplot,” and “survminer.”

## 3. Results

### 3.1. Clinical significance of MMP9

A total of 430 RCC patients were included from the TCGA-kidney renal clear cell carcinoma (KIRC) cohort. Using the R package “survminer,” the optimal cutoff value for MMP9 expression levels was determined to be 1.8501. Based on this threshold, patients were stratified into a high-expression group (n = 225) and a low-expression group (n = 205). Baseline characteristics are summarized in Table 1. Covariates such as age and sex showed no statistically significant distributional differences between the MMP9 high- and low-expression groups (*P* ≥ .05). In contrast, covariates including tumor histological grade and pathological staging demonstrated significant distributional differences between the 2 groups (*P* < .05).

**Table 1:** Characteristics of patients in the high-expression group and low-expression group of MMP9.

Variables	Total (n = 430)	Low (n = 205)	High (n = 225)	*P*
Age, n (%)				.221
~59	208 (48)	106 (52)	102 (45)	
60~	222 (52)	99 (48)	123 (55)	
Gender, n (%)				.634
Female	143 (33)	71 (35)	72 (32)	
Male	287 (67)	134 (65)	153 (68)	
Pathologic_stage, n (%)				.006
Stage I	213 (50)	118 (58)	95 (42)	
Stage II	42 (10)	21 (10)	21 (9)	
Stage III	105 (24)	41 (20)	64 (28)	
Stage IV	70 (16)	25 (12)	45 (20)	
Histologic_grade, n (%)				.005
G1/G2	189 (44)	105 (51)	84 (37)	
G3/G4	241 (56)	100 (49)	141 (63)	
Pharmaceutical_therapy, n (%)				.015
No	359 (83)	181 (88)	178 (79)	
Yes	71 (17)	24 (12)	47 (21)	
Laterality, n (%)				.798
Left	201 (47)	94 (46)	107 (48)	
Right	229 (53)	111 (54)	118 (52)	
Neoplasm_status, n (%)				.01
Tumor free	294 (68)	153 (75)	141 (63)	
With tumor	136 (32)	52 (25)	84 (37)	

MMP9 = matrix metalloproteinase-9.

**Figure 2.**
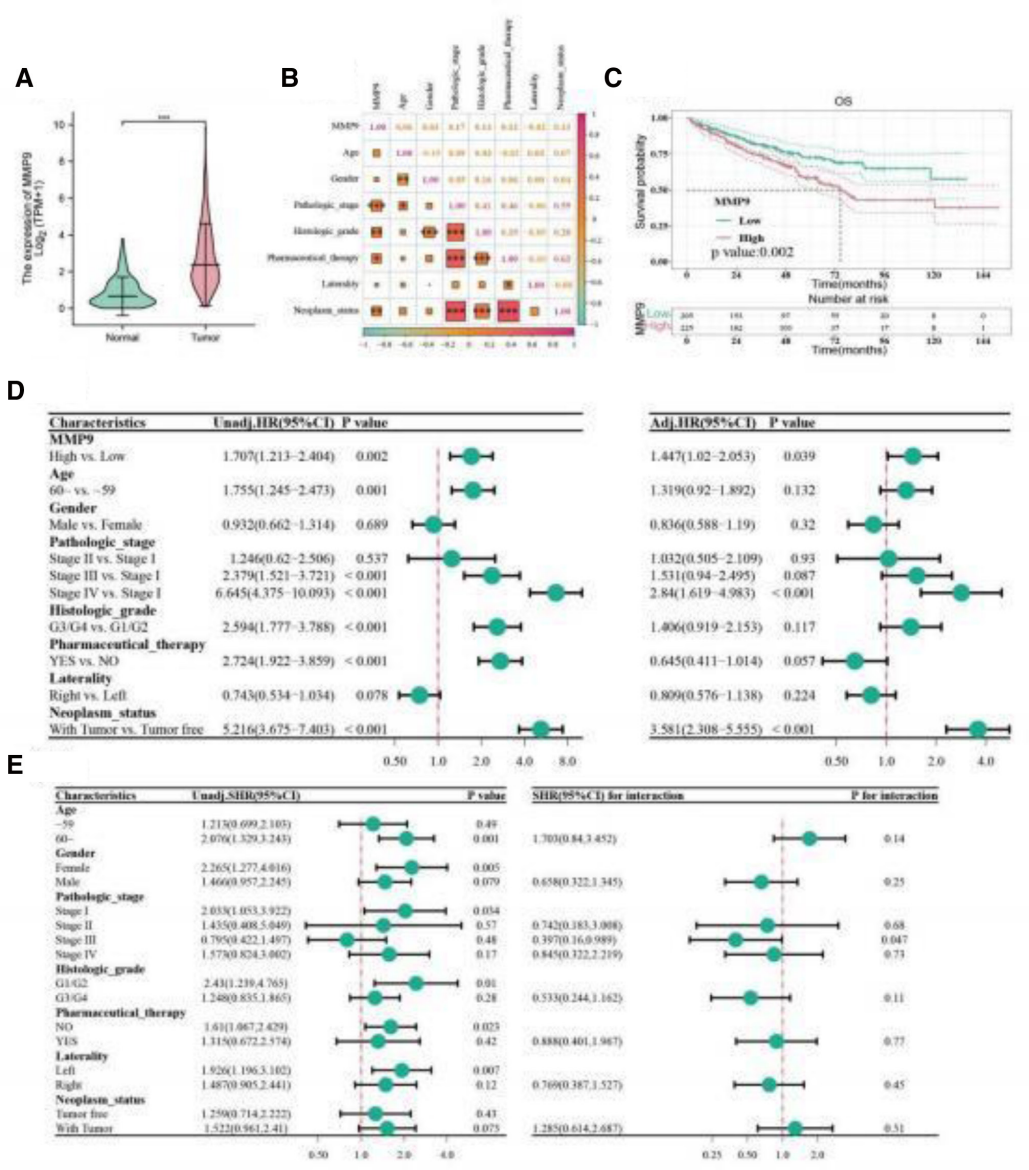
Differential expression analysis, correlation analysis, survival analysis, Cox proportional hazard model, subgroup analysis, and interaction test. (A) Differential expression analysis of MMP9 between normal and tumor groups. (B) Correlation heat maps between MMP9 and clinical parameters. (C) Kaplan–Meier analysis of the MMP9^high^ and MMP9^low^ groups. (D) Cox proportional hazards analysis. (E) Impact of MMP9 on patient prognosis across covariate-stratified subgroups. MMP9 = matrix metalloproteinase-9, OS = overall survival. ^ns^*P* *≥* .05; *P < .05; **P < .01; ***P < .001.

Differential analysis revealed that MMP9 expression levels in RCC tissue samples were significantly higher than in normal kidney tissues, with a median difference of 1.598 (95% CI = 1.356–1.868, *P* < .001; Fig. 2A). Furthermore, the correlation heatmap between MMP9 and clinical tumor characteristics demonstrated significant associations (*P* < .05) between the primary variable MMP9 and covariates including Pathologic_stage, Histologic_grade, Pharmaceutical_therapy, and Neoplasm_status (Fig. 2B).

Kaplan–Meier survival curves (Fig. 2C) indicated that the high-expression group had a median survival time of 74.7 months, whereas the low-expression group did not reach a median survival time due to an insufficient number of events. Elevated MMP9 expression was significantly associated with reduced OS in patients (*P* = .002). Univariate Cox regression analysis revealed that high MMP9 expression was significantly associated with poorer survival (HR = 1.707, 95% CI = 1.213–2.404, *P* = .002). Multivariate Cox regression further confirmed that high MMP9 expression served as an independent risk factor for OS in RCC patients (HR = 1.447, 95% CI = 1.020–2.053, *P* = .039; Fig. 2D). In addition, subgroup analysis showed that high MMP9 expression was significantly associated with poorer OS in patients aged ≥60 years (HR = 2.076, 95% CI = 1.329–3.243, *P* = .001), but not in those aged <60 years (HR = 1.213, 95% CI = 0.699–2.103, *P* = .49). The interaction test (*P* = .14) indicated no significant interaction between MMP9 and age (Fig. 2E). These findings collectively reveal the independent predictive value of MMP9 expression within the RCC cohort.

### 3.2. Exploring the potential mechanisms of MMP9 in RCC

**Figure 3.**
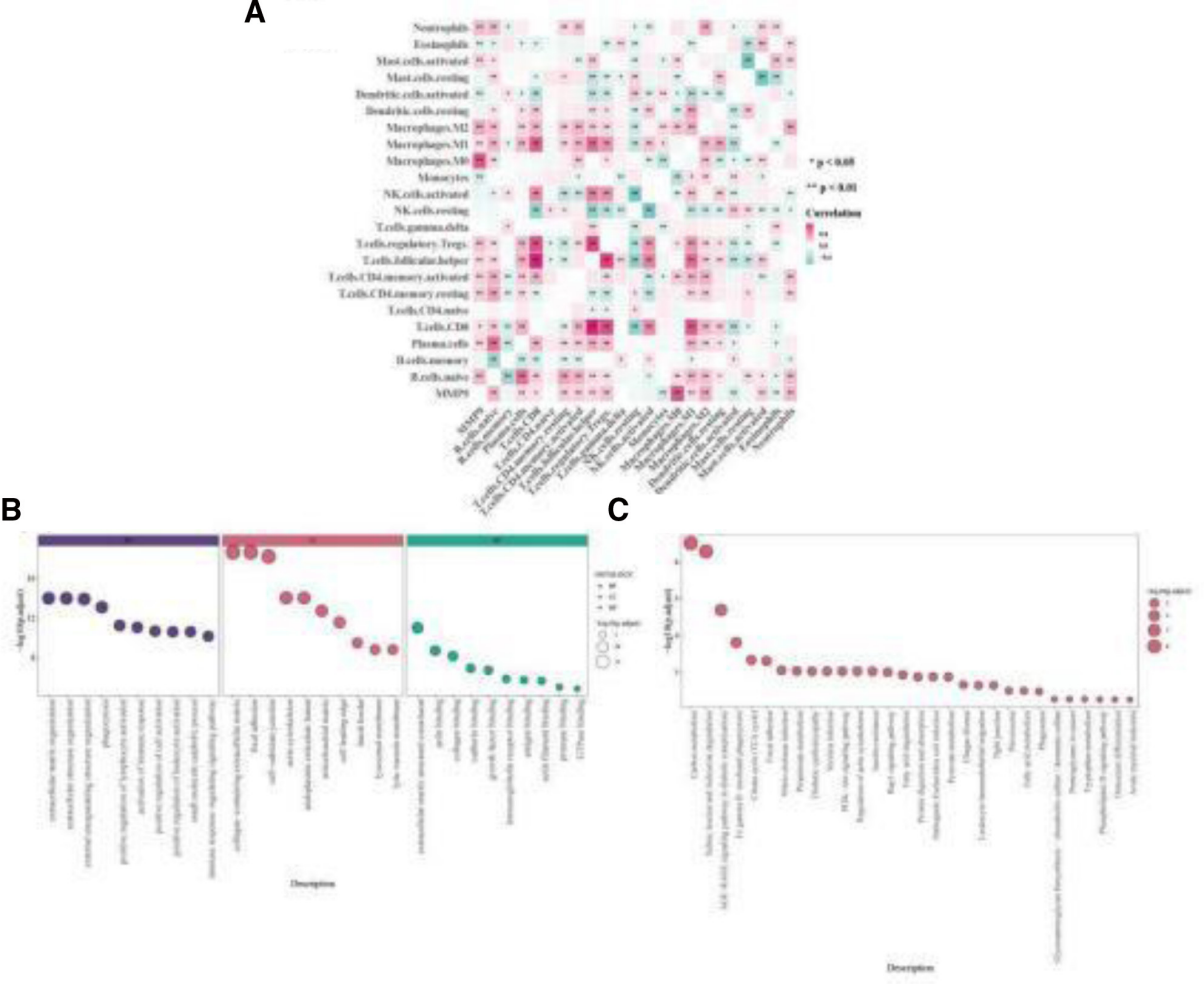
Immune cell infiltration (A), GO (B), and KEGG (C) enrichment analysis of differentially expressed genes between MMP9^high^ and MMP9^low^ groups. AGE-RAGE = advanced glycation end-products-receptor for advanced glycation end-products, GO = Gene Ontology, GTPase = guanosine triphosphatase, KEGG = Kyoto Encyclopedia of Genes and Genomes, MMP9 = matrix metalloproteinase-9, NK = natural killer cells, PI3K-Akt = phosphatidylinositol 3-kinase-Akt, Rap1 =Ras-associated protein 1, TCA = tricarboxylic acid, Tregs = regulatory T cells. ^ns^*P* ≥ .05; *P < .05; **P < .01; ***P < .001.

In RCC, immune infiltration analysis showed that high MMP9 expression was positively correlated with M2 macrophage infiltration (*r* = 0.32, *P* = 2.19 × 10^−11^). MMP9 expression was also significantly associated with multiple immune cell types, including plasma cells, CD8^+^ T cells, resting and activated memory CD4^+^ T cells, follicular helper T cells, M0/M1 macrophages, activated dendritic cells, mast cells, eosinophils, and neutrophils. These results suggest that MMP9 plays a multifaceted immunomodulatory role in RCC and may contribute to an immunosuppressive tumor microenvironment (TME) (Fig. 3A). GO enrichment analysis revealed that differentially expressed genes between MMP9 high- and low-expression groups were significantly enriched in BPs, including immune response regulation signaling pathways and GTPase binding. KEGG enrichment analysis indicated these differentially expressed genes were also significantly enriched in pathways such as carbon metabolism and PI3K-Akt signaling pathway (Fig. 3B, C).

### 3.3. Construction and evaluation of the radiomics model

**Figure 4.**
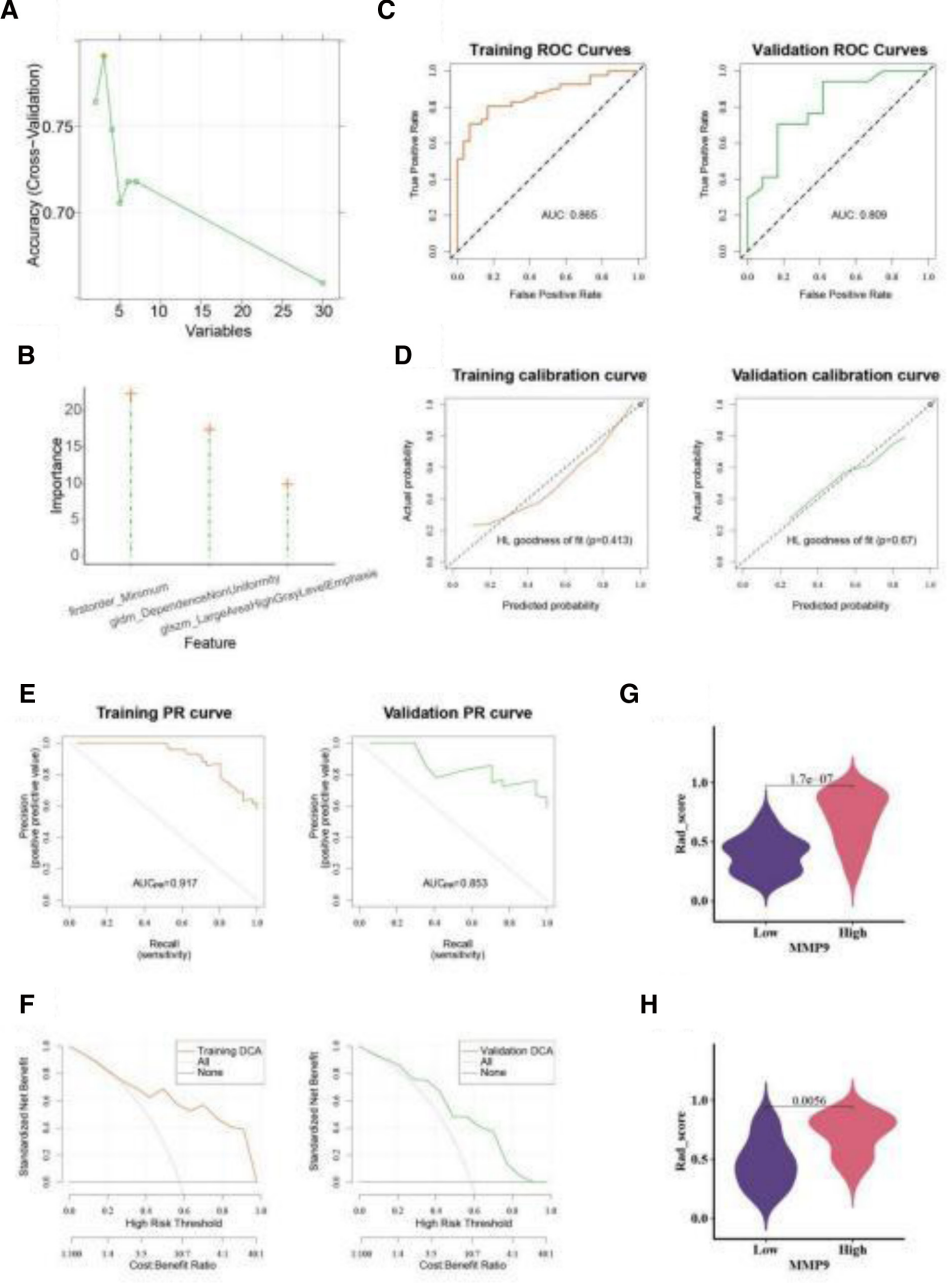
Construction and evaluation of radiologic model: (A) schematic diagram of the RFE screening process; (B) importance of filtered features in GBM algorithm; (C) ROC curves; (D) calibration curves; (E) PR curves; (F) DCA; violin plots of Rad-score in MMP9^high^ and MMP9^low^ groups in (G) the training set and (H) the validation set. DCA = decision curve analysis, MMP9 = matrix metalloproteinase-9, PR = precision-recall, Rad_score = radiomics score, RFE = recursive feature elimination, ROC = receiver operating characteristic.

A total of 100 RCC cases were included from the TCIA-CT database. Among the extracted radiomic features, approximately 95.3% exhibited ICCs ≥ 0.75, 3.7% had ICC values between 0.51 and 0.74, and 0.9% showed ICC values below 0.50. Following screening, 102 radiomic features with ICC ≥ 0.75 (out of 107 total features) were included for subsequent analysis. Patients were divided into a training set (n = 71) and a validation set (n = 29). Intergroup comparative analysis demonstrated balanced baseline characteristics between the 2 cohorts, indicating comparability (*P* > .05; Table 2). After screening the top 30 features using the minimum redundancy maximum relevance method, the recursive feature elimination algorithm was further employed to identify the optimal feature subset. Three features, including firstorder_Minimum, glszm_LargeAreaHighGrayLevelEmphasis, and gldm_DependenceNonUniformity, were selected for final model construction (Fig. 4A). The GBM algorithm was then used to develop the radiomics model, and the importance of the 3 features was ranked as shown in Figure 4B.

**Table 2:** Characteristics of patients in the training set and validation set for MMP9.

Variables	Total (n = 100)	Train (n = 71)	Validation (n = 29)	*P*
MMP9, n (%)				1
Low	42 (42)	30 (42)	12 (41)	
High	58 (58)	41 (58)	17 (59)	
Age, n (%)				.827
~59	62 (62)	45 (63)	17 (59)	
60~	38 (38)	26 (37)	12 (41)	
Gender, n (%)				.373
Female	36 (36)	28 (39)	8 (28)	
Male	64 (64)	43 (61)	21 (72)	
Pathologic_stage, n (%)				.105
Stage I	52 (52)	41 (58)	11 (38)	
Stage II	10 (10)	6 (8)	4 (14)	
Stage III	25 (25)	18 (25)	7 (24)	
Stage IV	13 (13)	6 (8)	7 (24)	
Histologic_grade, n (%)				.49
G1/G2	38 (38)	29 (41)	9 (31)	
G3/G4	62 (62)	42 (59)	20 (69)	
Pharmaceutical_therapy, n (%)				.191
No	82 (82)	61 (86)	21 (72)	
Yes	18 (18)	10 (14)	8 (28)	
Laterality, n (%)				.908
Left	44 (44)	32 (45)	12 (41)	
Right	56 (56)	39 (55)	17 (59)	
Neoplasm_status, n (%)				.137
Tumor free	74 (74)	56 (79)	18 (62)	
With tumor	26 (26)	15 (21)	11 (38)	
OS, n (%)				.294
0	68 (68)	51 (72)	17 (59)	
1	32 (32)	20 (28)	12 (41)	.044
OS time, median (Q1, Q3)	44.53 (23.1, 59.58)	49.5 (26.15, 60.72)	35.43 (14.43, 49.97)	

MMP9 = matrix metalloproteinase-9, OS = overall survival.

The GBM model demonstrated excellent predictive performance. In the training set, accuracy, specificity, sensitivity, positive predictive value, and negative predictive value were 0.803, 0.933, 0.707, 0.935, and 0.700, respectively. In the validation cohort, these metrics were 0.724, 0.750, 0.706, 0.800, and 0.643, respectively. Receiver operating characteristic curve analysis revealed AUC values of 0.865 (95% CI = 0.782–0.949) for the training set and 0.809 (95% CI = 0.645–0.973) for the validation set (Fig. 4C). The calibration curve and Hosmer-Lemeshow goodness-of-fit test indicated strong agreement between the predicted probabilities of high gene expression by this radiomics model and actual outcomes (*P* > .05; Fig. 4D). In the training set, the model achieved an area under the precision-recall curve of 0.917, while the test set area under the precision-recall curve was 0.853 (Fig. 4E). Decision curve analysis further demonstrated the model’s high clinical utility (Fig. 4F). The Delong test comparison of AUCs between training and validation sets showed no significant difference (*P* = .5494), indicating absence of overfitting or underfitting with consistent predictive performance across datasets. In addition, intergroup difference analysis revealed significantly distinct RS distributions between high- and low-expression groups in the training set (*P* < .01), where the MMP9 high-expression group exhibited elevated RS values (Fig. 4G, H).

### 3.4. Prognostic significance of the radiomics model

**Figure 5.**
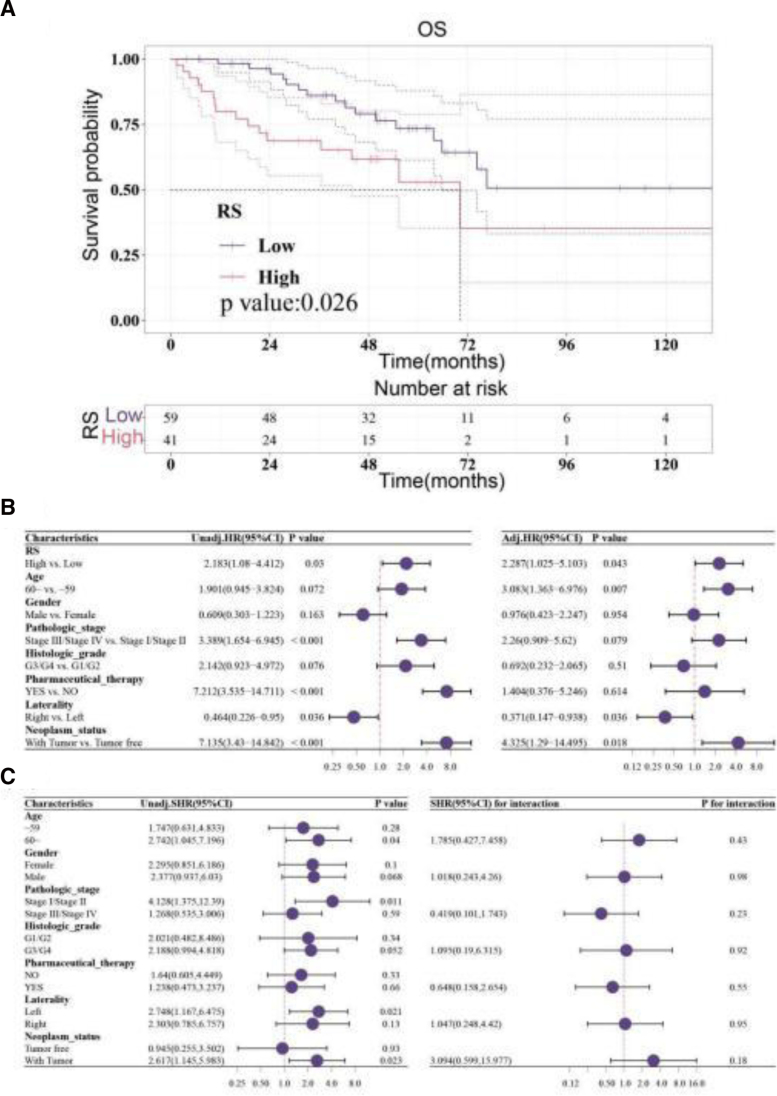
Survival analysis of TCGA-KIRC cohort intersected with TCIA cohort: (A) Kaplan–Meier curves of Rad_score groups; (B) Cox proportional hazards analysis; and (C) subgroup analysis and interaction test. KIRC = kidney renal clear cell carcinoma, OS = overall survival, RS = radiomics score, TCGA = The Cancer Genome Atlas, TCIA = The Cancer Imaging Archive.

Using an RS score cutoff value of 0.665, patients were stratified into high-RS (n = 41) and low-RS groups (n = 59). Except for pharmaceutical therapy (*P* = .029) and median OS time (*P* = .004), no significant differences were observed in the distribution of other covariates between the 2 groups (*P* > .05; Table 3). The median survival time was 70.17 months in the high-RS group, whereas it was not reached in the low-RS group due to fewer death events. Kaplan–Meier analysis revealed that high RS was significantly associated with poorer OS (*P* = .026; Fig. 5A). Univariate Cox regression analysis revealed that high RS was a significant risk factor for OS (HR = 2.183, 95% CI = 1.080–4.412, *P* = .03). After adjusting for confounding factors in multivariate Cox regression, high RS remained significantly associated with OS risk (HR = 2.287, 95% CI = 1.025–5.103, *P* = .043; Fig. 5B). Subgroup analysis demonstrated that RS was significantly associated with poorer OS in the patients aged ≥60 subgroup (HR = 2.742, 95% CI = 1.045–7.196, *P* = .04). Interaction analysis suggested a consistent prognostic value of RS across age groups (Fig. 5C). These findings demonstrate that the enhanced CT radiomics model score based on MMP9 expression exhibits robust independent prognostic value in RCC patients.

**Table 3:** Characteristics of patients in Rad-score^high^ and Rad-score^low^ groups.

Variables	Total (n = 100)	Low (n = 59)	High (n = 41)	*P*
Age, n (%)				.421
~59	62 (62)	39 (66)	23 (56)	
60~	38 (38)	20 (34)	18 (44)	
Gender, n (%)				.912
Female	36 (36)	22 (37)	14 (34)	
Male	64 (64)	37 (63)	27 (66)	
Pathologic_stage, n (%)				.221
Stage I/Stage II	62 (62)	40 (68)	22 (54)	
Stage III/Stage IV	38 (38)	19 (32)	19 (46)	
Histologic_grade, n (%)				.651
G1/G2	38 (38)	24 (41)	14 (34)	
G3/G4	62 (62)	35 (59)	27 (66)	
Pharmaceutical_therapy, n (%)				.029
No	82 (82)	53 (90)	29 (71)	
Yes	18 (18)	6 (10)	12 (29)	
Laterality, n (%)				.528
Left	44 (44)	28 (47)	16 (39)	
Right	56 (56)	31 (53)	25 (61)	
Neoplasm_status, n (%)				.075
Tumor free	74 (74)	48 (81)	26 (63)	
With tumor	26 (26)	11 (19)	15 (37)	
OS, n (%)				.3
0	68 (68)	43 (73)	25 (61)	
1	32 (32)	16 (27)	16 (39)	
OS time, median (Q1, Q3)	44.53 (23.1, 59.58)	49.77 (29.55, 64.73)	35.43 (13.33, 49.93)	.004

OS = overall survival, Rad_score = radiomics score.

## 4. Discussion

RCC, particularly clear cell RCC, is increasingly recognized as a metabolic disease characterized by profound reprogramming of energetic metabolism. Previous studies have shown that ccRCC exhibits altered glycolytic flux, impaired mitochondrial bioenergetics and oxidative phosphorylation, and dysregulated lipid metabolism. These metabolic alterations are closely associated with tumor progression, angiogenesis, immune remodeling, therapeutic resistance, and cancer stem cell-like properties. In the present study, KEGG enrichment analysis showed that genes differentially expressed between the MMP9 high- and low-expression groups were enriched in carbon metabolism and PI3K-Akt signaling pathways.^[18–21]^ These findings suggest that MMP9 may be involved not only in extracellular matrix remodeling but also in metabolic reprogramming-related tumor progression. MMP9-mediated matrix degradation, angiogenesis, inflammatory signaling, and immune cell recruitment may interact with altered metabolic pathways, thereby contributing to a more aggressive and therapy-resistant RCC phenotype. Therefore, the association between MMP9, metabolic reprogramming, and radiomic heterogeneity deserves further investigation.

We developed a radiomics model based on MMP9 expression to predict prognosis in RCC patients through the Rad-score (RS) and explored the underlying biological mechanisms of MMP9 using immune cell infiltration analysis and enrichment analysis. Key findings include: high MMP9 expression significantly correlated with OS in RCC patients (HR = 1.707, 95% CI = 1.213–2.404, *P* = .002), indicating its potential as an independent prognostic factor; high expression of MMP9 is not only significantly correlated with the infiltration of various immune cells, including M2 macrophages, CD8^+^ T cells, and dendritic cells, but its differentially expressed genes are also markedly enriched in the PI3K-Akt and immunomodulatory signaling pathways. These findings suggest that MMP9 may drive RCC progression by shaping an immunosuppressive TME; the prediction model constructed from 3 radiomic features demonstrated excellent discriminatory performance (AUC = 0.865), and the resulting RS served as an independent risk factor for RCC patients (HR = 2.287). Collectively, these results support the feasibility of integrating radiomics and transcriptomics for prognostic assessment in RCC and provide a potential framework for noninvasive risk stratification in clinical practice.

KIRC is considered one of the most immune-infiltrated malignancies, and the tumor immune microenvironment has a profound influence on disease biology, prognosis, and response to systemic therapy. Tumor-infiltrating immune cells, including CD8^+^ T cells, regulatory T cells, macrophages, dendritic cells, mast cells, and neutrophils, may contribute to both antitumor immunity and tumor-promoting inflammation depending on their functional states. In the present study, MMP9 expression was positively correlated with M2 macrophage infiltration and was also associated with multiple immune cell populations, suggesting that MMP9 may participate in remodeling the immune microenvironment of RCC. M2 macrophages generally exhibit immunosuppressive and pro-tumor properties and can promote extracellular matrix remodeling, angiogenesis, tumor cell invasion, and immune evasion. Therefore, the positive association between MMP9 expression and M2 macrophage infiltration may partly explain the poorer prognosis observed in patients with high MMP9 expression.^[22]^ In addition to immune infiltration, metabolic reprogramming and angiogenesis are central biological features of KIRC. Emerging evidence suggests that activation of specific metabolic pathways can regulate angiogenic and inflammatory signatures in RCC. In our enrichment analysis, genes differentially expressed between MMP9-high and MMP9-low groups were significantly enriched in carbon metabolism and PI3K-Akt signaling pathways. These findings indicate that MMP9 may be involved not only in extracellular matrix degradation but also in the interaction between metabolic remodeling, angiogenesis, and inflammatory signaling. Mechanistically, MMP9 can degrade extracellular matrix and basement membrane components, release matrix-bound pro-angiogenic factors, facilitate endothelial cell migration, and promote neovascularization. Meanwhile, MMP9 may modulate immune cell recruitment and inflammatory responses, thereby contributing to immunoflogosis and TME remodeling.^[23]^ Taken together, these findings suggest that MMP9 may serve as a key molecular link connecting metabolic reprogramming, angiogenesis, immune infiltration, and inflammation in RCC. These BPs may also provide a potential explanation for the predictive value of CT-derived radiomic features. The selected radiomic features may reflect intratumoral heterogeneity related to necrosis, hypoxia, heterogeneous enhancement, stromal remodeling, angiogenesis, and immune cell infiltration. Therefore, the MMP9-related RS may serve as a noninvasive imaging surrogate for TME complexity and biological aggressiveness. However, these mechanistic interpretations are exploratory and require further validation through radiology–pathology correlation, multiplex immunohistochemistry, spatial transcriptomics, and in vitro or in vivo functional experiments.^[24]^

The selected radiomic features may indirectly reflect these BPs. firstorder_Minimum may correspond to low-density regions such as necrosis, hypoxia, edema, or stromal remodeling, which may be associated with MMP9-mediated extracellular matrix degradation and microenvironmental alteration. glszm_LargeAreaHighGrayLevelEmphasis may reflect large high-enhancement regions related to vascularity or compact tumor cellularity, potentially corresponding to angiogenesis and PI3K-Akt-associated proliferation. gldm_DependenceNonUniformity may capture spatial heterogeneity caused by uneven cellular density, necrosis, immune infiltration, and stromal remodeling. Thus, the CT-based radiomics model may provide a noninvasive imaging surrogate for MMP9-related tumor heterogeneity and microenvironmental complexity. However, these interpretations remain exploratory and should be validated by radiology–pathology correlation and functional experiments.

Specifically, elevated MMP9 levels are significantly associated with higher histological grade, advanced clinical stage, and lymph node metastasis, serving as an independent prognostic factor in RCC.^[25]^ Furthermore, its expression is markedly higher in RCC tissues compared to normal renal tissues and tends to increase with tumor stage progression.^[26]^ The prognostic value of MMP9 has also been extensively validated in other malignancies. In breast cancer, high MMP9 expression correlates with patient age, TNM staging, tumor size, and lymph node metastasis, establishing it as a crucial indicator for prognostic assessment.^[27,28]^ In lung cancer, MMP9 not only shows significant associations with TNM staging, differentiation grade, and lymph node metastasis but also closely correlates with clinicopathological characteristics and 3-year survival rates.^[29]^ Furthermore, colorectal cancer,^[30]^ hepatocellular carcinoma,^[31]^ cervical cancer,^[32]^ prostate cancer,^[33]^ and gastric cancer^[34]^ have consistently demonstrated that high MMP9 expression correlates with enhanced tumor aggressiveness, shortened survival, and poor prognosis. Building upon this extensive evidence, our findings further validate MMP9 as an independent prognostic risk factor in RCC.

Functional enrichment analysis revealed elevated M2-type macrophage infiltration in the MMP9 high-expression group, with differentially expressed genes significantly enriched in immune-related pathways such as PI3K-AKT. This finding aligns with previous research, further substantiating that MMP9 may promote tumor progression by modulating the tumor immune microenvironment.^[35,36]^ Mechanistically, MMP9 facilitates tumor cell infiltration and metastasis by degrading the extracellular matrix and basement membrane, while also participating in tumor angiogenesis to provide necessary nutrients for tumor growth.^[37]^ Simultaneously, MMP9 regulates the immune microenvironment, influencing tumor immune evasion and treatment response.^[38]^ For instance, in RCC, high MMP9 expression correlates with increased infiltration of tumor-associated macrophages, potentially affecting prognosis by promoting the formation of an immunosuppressive microenvironment. Notably, M2-type macrophages possess immunosuppressive and pro-tumor properties, which may further reinforce the immunosuppressive state of the TME.^[39,40]^ Furthermore, activation of the PI3K/AKT signaling pathway can upregulate the mRNA and protein expression of its downstream target MMP9, thereby promoting the progression of RCC.^[41]^ Given the pro-tumor properties of M2-type macrophages and MMP9’s multifaceted cancer-promoting mechanisms, targeting MMP9 and its upstream signaling pathways (such as intervening in PI3K-AKT or blocking M2 polarization) may represent a crucial strategy for ameliorating the immunosuppressive microenvironment and enhancing the efficacy of current treatments.^[42]^ Overall, these findings highlight MMP9 as a key modulator of tumor immunity and a promising target for future therapeutic interventions in RCC.

Radiomic features can extract and quantify intratumoral heterogeneity information in a high-throughput manner, demonstrating significant value for assessing RCC prognosis. In this study, we identified 3 key radiomic features: firstorder_Minimum reflects the minimum grayscale value in images, potentially corresponding to necrotic or low-density areas within tumors; glszm_LargeAreaHighGrayLevelEmphasis emphasizes large high-intensity regions, suggesting dense tumor tissue or specific cellular aggregation patterns; and gldm_DependenceNonUniformity quantifies the nonuniformity of gray-level dependence relationships, potentially characterizing heterogeneity within the TME.^[43]^ Notably, studies have reported that RS models, constructed based on radiomic features reflecting tumor heterogeneity, can predict recurrence-free survival in RCC patients.^[44]^ Furthermore, research by Varghese et al demonstrated that radiomic features can effectively distinguish PD-L1 expression status in RCC patients.^[45]^ Similarly, this study achieved noninvasive prediction of MMP9 expression levels in RCC using radiomic features constructed from enhanced CT images. RS may capture tumor heterogeneity and biological aggressiveness, and therefore may be associated with disease severity and subsequent treatment decisions. Although we adjusted for pharmacotherapy and other clinical covariates in multivariate Cox regression, residual confounding cannot be completely excluded. Propensity score matching was not performed because of the limited sample size, as matching would further reduce statistical power. Future studies with larger cohorts should incorporate propensity score matching, inverse probability weighting, or other causal inference approaches to better control treatment-related confounding. The 3 selected radiomic features may provide indirect imaging reflections of MMP9-related BPs. firstorder_Minimum represents the minimum gray-level intensity within the tumor and may correspond to low-density regions, including necrosis, hypoxia, edema, or stromal remodeling. Because MMP9 participates in extracellular matrix degradation, basement membrane disruption, angiogenesis, and inflammatory cell recruitment, high MMP9 expression may contribute to microenvironmental remodeling and heterogeneous low-density areas on enhanced CT. glszm_LargeAreaHighGrayLevelEmphasis emphasizes large high-gray-level zones, which may reflect heterogeneous enhancement, vascular-rich regions, or compact tumor cellularity. This may be related to MMP9-mediated angiogenesis and PI3K-Akt-associated tumor proliferation. gldm_DependenceNonUniformity quantifies the nonuniformity of gray-level dependence and may capture spatial heterogeneity caused by variable cellular density, immune infiltration, necrosis, and stromal remodeling. Therefore, the selected features may collectively reflect MMP9-associated tumor heterogeneity and microenvironmental complexity. Nevertheless, these biological interpretations remain exploratory and require further validation by radiology–pathology correlation and functional experiments.

Compared with traditional detection methods, this study offers the following advantages: noninvasiveness: eliminates risks and discomfort associated with invasive procedures like biopsies; informational complementarity: integrates imaging features to capture tumor heterogeneity information that is difficult to detect through conventional methods; and translational potential: provides new insights for precision treatment of clear cell RCC (e.g., MMP9-targeted personalized therapies).

This study has several limitations. First, the sample size of the paired TCIA–TCGA cohort was relatively small, which may affect model stability and generalizability. Although the DeLong test showed no significant difference between the training and validation AUCs, this does not completely exclude the risk of sampling variation or overfitting under small-sample conditions. Second, this was a retrospective study, and selection bias cannot be fully avoided. Only patients with available imaging, transcriptomic, and clinical survival data were included, which may limit the representativeness of the cohort. Third, technical heterogeneity should be considered. TCIA contains multicenter imaging data, and differences in CT scanning protocols, reconstruction algorithms, slice thickness, tube voltage, and contrast-enhancement parameters may influence radiomic features. Although ICC screening reduced inter-observer segmentation variability, it could not eliminate heterogeneity caused by acquisition protocols. Fourth, the optimal cutoff values for MMP9 expression and RS were determined based on the current dataset, and their applicability to external cohorts remains uncertain. Fifth, pharmacotherapy status may be associated with disease severity and treatment decision-making. Although multivariate Cox regression was performed, residual confounding cannot be completely excluded, and PSM was not conducted because of the limited sample size. Finally, the mechanistic exploration of MMP9 was based mainly on bioinformatics analyses. Further in vitro and in vivo experiments are required to validate the causal role of MMP9 in metabolic reprogramming, immune infiltration, angiogenesis, and RCC progression.

Although the DeLong test showed no significant difference in AUC between the training and validation cohorts, this result should be interpreted cautiously because of the relatively small sample size. In small datasets, model performance may be influenced by sampling variation and may not fully reflect the stability of the model in broader clinical populations. Therefore, the current findings should be regarded as preliminary, and further external validation using larger, multicenter prospective cohorts is required to confirm the robustness and generalizability of the proposed radiomics model.

In conclusion, this study systematically analyzed the prognostic value of MMP9 in RCC and established a radiomics-based prediction model, providing a novel tool for patient prognosis management. In personalized treatment, this model can complement traditional indicators and is expected to assist clinical decision-making while improving patients’ quality of life. Future work should focus on promoting the clinical translation of the model and thoroughly exploring the feasibility of MMP9-targeted therapeutic strategies, ultimately enhancing precision diagnosis and treatment for RCC.

## 5. Conclusion

MMP9 expression levels served as a robust independent risk factor for RCC patients. The radiomics model constructed from enhanced CT-based imaging features enables noninvasive prediction of MMP9 expression levels and serves as an independent prognostic biomarker for RCC patients, providing novel approaches and tools for personalized treatment and more precise prognostic assessment.

## Author contributions

**Conceptualization:** Sijia Sun, Jin Zhang.

**Data curation:** Sijia Sun, Jin Zhang.

**Formal analysis:** Sijia Sun, Jin Zhang.

**Writing – original draft:** Sijia Sun, Jin Zhang.

**Writing – review & editing:** Sijia Sun, Jin Zhang.

**Funding acquisition:** Jin Zhang.

**Investigation:** Jin Zhang.

## References

[1] Siegel RL Miller KD Fuchs HE Jemal A. Cancer statistics, 2022. CA Cancer J Clin. 2022;72:7–33. 35020204 10.3322/caac.21708

[2] Rose TL Kim WY. Renal cell carcinoma. JAMA. 2024;332:1001–10. 39196544 10.1001/jama.2024.12848 PMC11790279

[3] Young M Jackson-Spence F Beltran L. Renal cell carcinoma. Lancet (Lond). 2024;404:476–91. 10.1016/S0140-6736(24)00917-6 39033764

[4] Leung DK Wong CH Ko IC. Global trends in the incidence, mortality, and risk-attributable deaths for prostate, bladder, and kidney cancers: a systematic analysis from the global burden of disease study 2021. Eur Urol Oncol. 2025;8:1533–43. 40441940 10.1016/j.euo.2025.05.007

[5] Bukowski RM. Natural history and therapy of metastatic renal cell carcinoma: the role of interleukin-2. Cancer. 1997;80:1198–220. 9317170 10.1002/(sici)1097-0142(19971001)80:7<1198::aid-cncr3>3.0.co;2-h

[6] Padala SA Barsouk A Thandra KC. Epidemiology of renal cell carcinoma. World J Oncol. 2020;11:79–87. 32494314 10.14740/wjon1279 PMC7239575

[7] Bahadoram S Davoodi M Hassanzadeh S Bahadoram M Barahman M Mafakher L. Renal cell carcinoma: an overview of the epidemiology, diagnosis, and treatment. G Ital Nefrol. 2022;39:2022-vol3. 35819037

[8] Chen Y-W Wang L Panian J. Treatment landscape of renal cell carcinoma. Curr Treat Options Oncol. 2023;24:1889–916. 38153686 10.1007/s11864-023-01161-5 PMC10781877

[9] Chen JF Ye SZ Wang KJ. Long non-coding RNA OSTM1-AS1 promotes renal cell carcinoma progression by sponging miR-491-5p and upregulating MMP-9. Sci Rep. 2025;15:359. 39747324 10.1038/s41598-024-83154-4 PMC11696353

[10] Zhang Q Ni Y Wang S. G6PD upregulates cyclin E1 and MMP9 to promote clear cell renal cell carcinoma progression. Int J Med Sci. 2022;19:47–64. 34975298 10.7150/ijms.58902 PMC8692124

[11] Li K Li D Hafez B. Identifying and validating MMP family members (MMP2, MMP9, MMP12, and MMP16) as therapeutic targets and biomarkers in kidney renal clear cell carcinoma (KIRC). Oncol Res. 2024;32:737–52. 38560573 10.32604/or.2023.042925 PMC10972725

[12] Kang W Qiu X Luo Y. Application of radiomics-based multiomics combinations in the tumor microenvironment and cancer prognosis. J Transl Med. 2023;21:598. 37674169 10.1186/s12967-023-04437-4 PMC10481579

[13] Fan X Li J Huang B. Noninvasive radiomics model reveals macrophage infiltration in glioma. Cancer Lett. 2023;573:216380. 37660885 10.1016/j.canlet.2023.216380

[14] Vivian J Rao AA Nothaft FA. Toil enables reproducible, open source, big biomedical data analyses. Nat Biotechnol. 2017;35:314–6. 28398314 10.1038/nbt.3772 PMC5546205

[15] Xu S Hu E Cai Y. Using clusterProfiler to characterize multiomics data. Nat Protoc. 2024;19:3292–320. 39019974 10.1038/s41596-024-01020-z

[16] Newman AM Steen CB Liu CL. Determining cell type abundance and expression from bulk tissues with digital cytometry. Nat Biotechnol. 2019;37:773–82. 31061481 10.1038/s41587-019-0114-2 PMC6610714

[17] Kiss FJ Jaro AI Mathe D. Predicting PD-L1 expression status in NSCLC using radiomic analysis of ^18^F-FDG-PET/CT images. Eur J Nucl Med Mol Imaging. 2025;53:800–11. 40668270 10.1007/s00259-025-07453-2 PMC12830409

[18] Lucarelli G Lasorsa F Milella M. Transcriptomic and proteo-metabolic determinants of the grading system in clear cell renal cell carcinoma. Urol Oncol. 2025;43:469.e19–32. 10.1016/j.urolonc.2025.02.016 40082108

[19] di Meo NA Lasorsa F Rutigliano M. The dark side of lipid metabolism in prostate and renal carcinoma: novel insights into molecular diagnostic and biomarker discovery. Expert Rev Mol Diagn. 2023;23:297–313. 36960789 10.1080/14737159.2023.2195553

[20] Lucarelli G Loizzo D Franzin R. Metabolomic insights into pathophysiological mechanisms and biomarker discovery in clear cell renal cell carcinoma. Expert Rev Mol Diagn. 2019;19:397–407. 30983433 10.1080/14737159.2019.1607729

[21] Di Meo NA Lasorsa F Rutigliano M. Renal cell carcinoma as a metabolic disease: an update on main pathways, potential biomarkers, and therapeutic targets. Int J Mol Sci . 2022;23:14360. 36430837 10.3390/ijms232214360 PMC9698586

[22] Vuong L Kotecha RR Voss MH Hakimi AA. Tumor microenvironment dynamics in clear-cell renal cell carcinoma. Cancer Discov. 2019;9:1349–57. 31527133 10.1158/2159-8290.CD-19-0499 PMC6774890

[23] Gigante M Pontrelli P Herr W. miR-29b and miR-198 overexpression in CD8+ T cells of renal cell carcinoma patients down-modulates JAK3 and MCL-1 leading to immune dysfunction. J Transl Med. 2016;14:84. 27063186 10.1186/s12967-016-0841-9 PMC4827202

[24] Lasorsa F Rutigliano M Milella M. Complement system and the kidney: its role in renal diseases, kidney transplantation and renal cell carcinoma. Int J Mol Sci. 2023;24:16515. 38003705 10.3390/ijms242216515 PMC10671650

[25] Zeng Y Gao M Lin D Du G Cai Y. Prognostic and immunological roles of MMP-9 in pan-cancer. Biomed Res Int. 2022;2022:2592962. 35178444 10.1155/2022/2592962 PMC8844435

[26] Mlynarczyk G Gudowska-Sawczuk M Mroczko B Bruczko-Goralewska M Romanowicz L Tokarzewicz A. Higher content but no specific activity in gelatinase B (MMP-9) compared with gelatinase A (MMP-2) in human renal carcinoma. Cancers (Basel). 2023;15:5475. 38001735 10.3390/cancers15225475 PMC10670015

[27] Jiang H Li H. Prognostic values of tumoral MMP2 and MMP9 overexpression in breast cancer: a systematic review and meta-analysis. BMC Cancer. 2021;21:149. 33568081 10.1186/s12885-021-07860-2 PMC7877076

[28] Yuan J Xiao C Lu H. Effects of various treatment approaches for treatment efficacy for late stage breast cancer and expression level of TIMP-1 and MMP-9. Cancer Biomark. 2018;23:1–7. 30010105 10.3233/CBM-170901 PMC13078551

[29] Zhang H Zhao B Zhai ZG Zheng J-D Wang Y-K Zhao Y-Y. Expression and clinical significance of MMP-9 and P53 in lung cancer. Eur Rev Med Pharmacol Sci. 2021;25:1358–65. 33629306 10.26355/eurrev_202102_24844

[30] Hong XC Liang QL Chen M. PRL-3 and MMP9 expression and epithelial-mesenchymal transition markers in circulating tumor cells from patients with colorectal cancer: potential value in clinical practice. Front Oncol. 2022;12:878639. 35574414 10.3389/fonc.2022.878639 PMC9104807

[31] Cui Q Wang X Zhang Y Shen Y Qian Y. Macrophage-derived MMP-9 and MMP-2 are closely related to the rupture of the fibrous capsule of hepatocellular carcinoma leading to tumor invasion. Biol Proced Online. 2023;25:8. 36918768 10.1186/s12575-023-00196-0 PMC10012540

[32] Chen W Huang S Shi K Yi L Liu Y Liu W. Prognostic role of matrix metalloproteinases in cervical cancer: a meta-analysis. Cancer Control. 2021;28:10732748211033743. 34482737 10.1177/10732748211033743 PMC8424604

[33] Pinheiro LCL Pereira R Francelino AL. Metalloproteinase 9 immunostaining profile is positively correlated with tumor grade, extraprostatic extension and biochemical recurrence in prostate cancer. Pathol Res Pract. 2024;253:155024. 38113764 10.1016/j.prp.2023.155024

[34] Shang W Wang Y Liang X. SETDB1 promotes gastric carcinogenesis and metastasis via upregulation of CCND1 and MMP9 expression. J Pathol. 2021;253:148–59. 33044755 10.1002/path.5568

[35] Hong Y Lv Z Xing Z. Identification of molecular subtypes and diagnostic model in clear cell renal cell carcinoma based on collagen-related genes may predict the response of immunotherapy. Front Pharmacol. 2024;15:1325447. 38375034 10.3389/fphar.2024.1325447 PMC10875022

[36] Qiu Y Liao Y Zhang J. Exploring the role of coagulation-related genes in renal cell carcinoma: implications for tumor microenvironment and prognostic biomarkers. Comput Biol Chem. 2024;110:108082. 38663187 10.1016/j.compbiolchem.2024.108082

[37] Augoff K Hryniewicz-Jankowska A Tabola R Stach K. MMP9: a tough target for targeted therapy for cancer. Cancers (Basel). 2022;14:1847. 35406619 10.3390/cancers14071847 PMC8998077

[38] Kusmartsev S. Metastasis-promoting functions of myeloid cells. Cancer Metastasis Rev. 2025;44:61. 40663210 10.1007/s10555-025-10278-y PMC12263759

[39] Xu Y Li L Yang W. TRAF2 promotes M2-polarized tumor-associated macrophage infiltration, angiogenesis and cancer progression by inhibiting autophagy in clear cell renal cell carcinoma. J Exp Clin Cancer Res. 2023;42:159. 37415241 10.1186/s13046-023-02742-w PMC10324183

[40] Braun DA Street K Burke KP. Progressive immune dysfunction with advancing disease stage in renal cell carcinoma. Cancer Cell. 2021;39:632–48.e8. 33711273 10.1016/j.ccell.2021.02.013 PMC8138872

[41] Yue Y Hui K Wu S. MUC15 inhibits cancer metastasis via PI3K/AKT signaling in renal cell carcinoma. Cell Death Dis. 2020;11:336. 32382053 10.1038/s41419-020-2518-9 PMC7205982

[42] Karmokar PF Moniri NH. Free-fatty acid receptor-4 (FFA4/GPR120) differentially regulates migration, invasion, proliferation and tumor growth of papillary renal cell carcinoma cells. Biochem Pharmacol. 2023;213:115590. 37201877 10.1016/j.bcp.2023.115590

[43] Van Griethuysen JJM Fedorov A Parmar C. Computational radiomics system to decode the radiographic phenotype. Cancer Res. 2017;77:e104–e7. 29092951 10.1158/0008-5472.CAN-17-0339 PMC5672828

[44] Yang G Nie P Yan L. The radiomics-based tumor heterogeneity adds incremental value to the existing prognostic models for predicting outcome in localized clear cell renal cell carcinoma: a multicenter study. Eur J Nucl Med Mol Imaging. 2022;49:2949–59. 35344062 10.1007/s00259-022-05773-1

[45] Varghese B Cen S Zahoor H. Feasibility of using CT radiomic signatures for predicting CD8-T cell infiltration and PD-L1 expression in renal cell carcinoma. Eur J Radiol Open. 2022;9:100440. 36090617 10.1016/j.ejro.2022.100440 PMC9460152

